# Variation of RNA Quality and Quantity Are Major Sources of Batch Effects in Microarray Expression Data

**DOI:** 10.3390/microarrays3040322

**Published:** 2014-12-16

**Authors:** Mario Fasold, Hans Binder

**Affiliations:** 1 Interdisciplinary Centre for Bioinformatics, Universität Leipzig, Härtelstr. 16–18, 04107 Leipzig, Germany; E-Mail: binder@izbi.uni-leipzig.de; 2 ecSeq Bioinformatics, Brandvorwerkstrasse 43, 04275 Leipzig, Germany; 3 Leipzig Research Center for Civilization Diseases, Universität Leipzig, Philipp-Rosenthal-Straße 27, 04103 Leipzig, Germany

**Keywords:** microarray, batch effects, expression analysis, quality control, RNA

## Abstract

The great utility of microarrays for genome-scale expression analysis is challenged by the widespread presence of batch effects, which bias expression measurements in particular within large data sets. These unwanted technical artifacts can obscure biological variation and thus significantly reduce the reliability of the analysis results. It is largely unknown which are the predominant technical sources leading to batch effects. We here quantitatively assess the prevalence and impact of several known technical effects on microarray expression results. Particularly, we focus on important factors such as RNA degradation, RNA quantity, and sequence biases including multiple guanine effects. We find that the common variation of RNA quality and RNA quantity can not only yield low-quality expression results, but that both factors also correlate with batch effects and biological characteristics of the samples.

## 1. Introduction

High-throughput transcriptome profiling is an essential technique in biomedical research. Countless gene expression studies have been performed using various available microarray platforms, including large-scale and meta studies comprising hundreds of experiments. The latter are enabled by the large and ever-growing number of samples archived in public data repositories. For example, about 250,000 new microarray samples have been publicly released and indexed by the ArrayExpress database [1] each year since 2010.

The general aim of gene expression experiments is to find systematic expression changes relating to variations in biological or environmental conditions. Systematic expression changes due to technical factors, however, constitute a bias that can negatively affect the reliability of the expression results. Technical factors often change between groups of samples (“batches”) where the technical variables, for example, could be that two different lab workers handle the sample groups, or that the experiments are carried out at different dates or locations.

These batch effects are a major issue in microarray data analysis, and correlation of such factors with the biological variables of interest can prevent identification of the true biological source of variation and render the results of a microarray experiment worthless [2]. An anecdotal example for this is the following case. Akey *et al.* reanalyzed a large microarray-based study intending to assess gene expression variation between human populations [3]. They found that the largest expression changes were not between the populations but rather between groups of samples processed at different time points—79% of genes were found to be differentially expressed between processing years but within the same population. This large amount of variation can hardly be reasonably explained by biology. Akey *et al.* concluded that the data possesses a systematic and confounding technical bias, and that the reliability of the obtained results is at least questionable.

In face of the widespread occurrence and the negative impact on the reliability of the outcome, it raises the question about the origins of batch effects. Currently they are often handled merely as a statistical issue (*i.e.*, as an unspecified source of variation) that can be detected within experimental data using a hypothesis- and thus model-based approach [4]. To identify and study the various sources of batch effects, together with their prevalence and their impact, is as important as it is challenging. One can assess the presence of a batch effect in a data set by testing for correlations between a potentially confounding factor and the expression measurements, but this requires that one has information on the factors potentially varying between the groups of samples. In practice, only very few of those factors are recorded in the course of an experiment, and one is often left with no more than the experimental date or location. In lack of other meta-data, which ideally should have been stored alongside the experiment, date and location of measurement are frequently used as surrogate variables for the assessment of batch effects.

The causal “true” sources of technical variation, however, are more likely to relate to other factors such as the specifics of the used hybridization buffers and instruments, or the quality of the amplified RNA. Some of these experimental factors can be assessed by relying on available primary data—raw probe intensities in the case of microarrays. Making use of the particular design of the devices and protocols indicators of technical properties of a specific microarray hybridization can be extracted from the primary data [5].

We here aim to investigate the general prevalence and the impact on the expression results for a number of technical factors using a large and representative set of microarray samples. We will rely on the common Affymetrix HG-U133a platform, focusing on variations of RNA quality, RNA quantity and sequence effects, which we suspect to constitute potential sources of batch effects. These technical factors are usually expected to be (a) constant in an ideal experiment and (b) largely independent of the biological variation of interest. Consequently, specific metrics for these factors represent covariates potentially explaining the unwanted technical differences in the expression results. We will not consider general quality-control (qc) metrics such as GNUSE [6] which are well suited to detect low quality microarray samples in general.

Our analysis uses the *HumanArraySet* representing a large number of publicly available microarray samples of this platform (see also Methods Section). After strict quality-control, 5372 of these 8131 samples have been selected for the *HumanExpressionAtlas*, a large collection of human gene expression measurements that can be queried via the ArrayExpress web service [7,8]. This data allows us to investigate technical parameters in sample sets representative for human microarray experiments and in subsets either passing (qc-included) or failing (qc-excluded) quality control. Furthermore, this allows studying the impact of the technical factors on the expression data of the gene expression atlas. Particularly, we focus on quality metrics characterizing RNA degradation, RNA quantity, and sequence biases including multiple guanine effects. We further estimated effect size: We address the important question about the consequences, *i.e.*, how strong the impact of the common variation among these factors is on the results of large-scale gene expression studies.

## 2. Methods

### 2.1. Human Expression Data

We have downloaded the *HumanExpressionAtlas* data set (E-MTAB-62 on Array Express) compiled by Lukk *et al.* [7] consisting of 5372 (“qc-included”) samples hybridized to Affymetrix HG-U133a microarrays. This data set comprises in total 206 different studies from 163 different laboratories. Using text mining and manual curation, each sample was assigned one of 369 biological groups representing distinct human cell and tissue types, disease states and cell lines. The resulting *expression space*, the combined and processed gene expression data from this diverse collection of human samples, can be queried using the dedicated database ArrayExpress Atlas [8].

The 5372 samples have been selected from a larger data set of 8268 samples after application of strict quality control (qc). This was based on the quality measures *scaling factor*, *average background*, *percentage of present calls*, *RNA degradation from whole array*, *Normalized Unscaled Standard Errors*, and *Relative Log Expression* computed from the array data using Bioconductor [9]. Quality thresholds were selected based on the recommendations given in [10] adjusted to the distribution of the quality measures within this data set. Most of these metrics are typically the first choice for judging whether the samples have or have not sufficient quality relative to the complete set, while however being quite unspecific for any particular technical effect.

We obtained a full list of the 8268 samples from the authors and downloaded the remaining 2896 (“qc-excluded”) samples from public databases. From these, 137 samples could however not be retained as they were removed from the databases, leaving in total 8268 − 137 = 8131 samples. The full set of 8268 unique samples represents virtually all HG-U133a data publicly available in the two major public databases GEO and ArrayExpress in 2006 with no restrictions on the type of samples. This *HumanArraysSet* therefore is a representative sample of available human microarray experiments.

### 2.2. Chip Characteristics Obtained from Physico-Chemical Modeling of Surface Hybridization

The key process in any microarray experiment is the hybridization of nucleic acids on a solid surface. Modeling this process based on physico-chemical principles has helped to improve the understanding of microarray surface hybridization and has been applied many times to precisely describe the response function of microarray probes [11,12,13,14,15]. Particularly, our previous work on microarray data analysis provided a comprehensive treatment of the physico-chemical processes involved in the hybridization and washing of microarrays, including the effects of non-specific binding, bulk hybridization and probe and target molecule interactions to develop practical algorithms for inferring target concentrations as expression measures (see [16] for an overview).

In the first step of our approach we disentangled the complex nature of microarray hybridization process by addressing a series of effects in separate studies in order to understand their nature, to establish causal relations between experimental factors and microarray measurements, to judge the effect size, and finally to develop models and algorithms that allow suitable calibration of the raw data. Particularly, we studied the character of the hybridization isotherm [17,18,19,20], the relation between the levels of non-specific background, specific hybridization and saturation [21], the effect of washing [13], the effect of target depletion due to surface hybridization [22], the effect of probe sequence including special sequence motifs [23,24,25] and of RNA-quality [26]. We developed physical models of surface hybridization and applied them to selected data sets, which specifically and systematically vary only one of the experimental factors (e.g., amount of RNA, its quality or the extent of post-hybridization washing) leaving all the other factors unchanged. This way we established causal relations and could describe each of the effects quantitatively in terms of well-defined measures using appropriate mathematical frameworks.

In the next step we developed practical algorithms such as the “Hook Curve” formalism [5,27] to estimate specific target abundances based on Langmuir-like models using only the information available in a given microarray experiment. It combines hybridization data of pairs of probes that bind the same transcript with different affinities such as the perfect match/mismatch (PM/MM) probe pairs on microarrays of the GeneChip-type. The plot of the log–intensity difference *versus* the logged mean intensity of these probe pairs provides curves of characteristic hook-like shape, the dimensions of which enable parameterization of the Langmuir isotherm in a chip- and probe-specific fashion and finally allow to extract expression measures from the data.

Moreover, the dimensions of the hook-curve, e.g., its starting point, its width and height, provide quality measures characterizing the hybridization of each particular array in terms of, e.g., the amount of RNA and the fractions of specific and non-specific hybridization in large scale experimental series. The hook approach was also adapted to assess RNA-quality using microarray data [26]. Here, probe pairs with different distances to the 3'-end of the transcripts were used for mutual referencing. It was demonstrated that decomposition of the probe signals into contributions due to specific and non-specific hybridization and consideration of saturation behavior might be essential for proper quality control of the RNA. The hook-approach also allows distinguishing between different hybridization mechanisms such as local and global depletion of targets in supernatant solution [22] and to identify different effects causing chip-to-chip intensity variance such as scanner settings or non-specific background levels (see [16] for details).

We applied the hook approach for quality control and the identification of batch effects and of outlier samples in large scale cancer data sets [28,29]. For inspection of a detailed quality report based on our hook parameters we refer to the supplement of [29]. In our previous work we also compared the performance of different generations of GeneChip arrays with respect to sequence effects and hybridization parameters [5,25] and of different preprocessing algorithms and with respect to the obtained expression measures [5,25] and derived biological interpretations [29].

As mentioned above, the hook-quality measures make use of internal standards available for arrays of the GeneChip-type such as the PM/MM pairs and sets of several probes interrogating different positions of the gene of interest. Due to the special design of these arrays we were able to monitor the hybridization characteristics of large experimental series. In this publication we focus on arrays of the Affymetrix HG-133 design (including, e.g., HG-U133A and B, HG-U133plus 2 and the respective plate arrays). For comparisons between GeneChip arrays of different generations we refer to our previous work [5,25]. It has been shown that the hybridization effects in general apply to all array types studied but their amplitude can vary considerably.

Importantly, the study of hybridization artifacts of “non-Affymetrix” microarrays, e.g., of the Illumina or Agilent products, would require first the development of appropriate methods of quality control which estimate analogous effects as discussed here. In general, we expect gradual differences in effect size but no principal difference due to the common working principle of microarrays based on sequence-specific surface hybridization [30]. Also the particular preprocessing method used for calibration can affect the amplitude of technical artifacts since different methods remove parasitic effects such as the non-specific background intensity or sequence effects of the intensity with different efficiency [5,25].

### 2.3. Positional-Dependent Sequence Model and Sequence Effect Size

The intensity of a microarray probe is modeled in dependency of specific/non-specific target concentration, saturation intensity and a sequence effect δA as described in [27]. We employ a positional-dependent nearest neighbor model describing the sequence effect as the sum of sensitivity terms over all 16 dinucleotide subsequences ξ^k,k+1 ^∈ {A, C, G, T}^2^ and all positions k = 1...24 of the 25-meric probe sequence ξ [17]

(1) $$\delta A(\xi )=\sum_{K=1}^{24}\sigma_{k}(\xi^{k,k+1})$$

The sensitivity profiles were calculated using the non-specific hybridization signal of all PM probes of the arrays (see Supplemental Figure S2 for an example).

We define the *maximum sensitivity amplitude* as the maximum difference between all pairs of NN-sensitivity profiles

(2) $$\text{log}(K_{\text{diff}})\equiv {\delta A}_{\text{max all ξ}}-{\delta A}_{\text{min all ξ}}$$

of the non-specific hybridization mode. It determines how much (in units of log intensity contributions) a probe could shine brighter than another one given that both probes target the same transcript and thus it estimates the sequence effect size.

### 2.4. Metrics of RNA Quantity

In [27] we define the relative hybridization degree, or S/N ratio,

(3) $$R_{p}\equiv \frac{S_{p}}{N_{p}}$$

as the level of specific hybridization S_p_ of a probe set *p* relative to the baseline of non-specific binding N_p_ of this probe set. Averaging these *R* values over all probe sets of a microarray exceeding a given expression threshold provides the *relative specific transcript level*, or mean log S/N ratio,

(4) $$\lambda ={\langle \text{log}(R+1)\rangle }_{R>0.5;{\text{chip}}^{\cdot }}$$

We further defined β as the mean negative decadic logarithm of the non-specific signal of all probe sets of the array

(5) $$\beta \approx -{\langle {\text{logN}}_{p}\rangle }_{{\text{chip}}^{\cdot }}$$

It was previously shown that β has a geometric interpretation as the width of the hook curve and describes the measuring range of specific hybridization [5].

## 3. Results and Discussion

### 3.1. Assessing RNA Quality

Good RNA quality is an important prerequisite for obtaining reliable results from a microarray gene expression experiment. RNA quality propagates from the hybridized sample to the obtained gene expression estimates and consequently also to differential expression results. Combined with the previous detection of a noticeable degradation effect upon the majority of microarray samples [31], variability of RNA quality has a high risk of being a major source of technical bias.

The RNA Integrity Number (RIN) provides a measure for RNA quality that is determined for most microarray samples before hybridization [32]. RIN values scale between 1 and 10, and using only samples with RIN ≥ 7 is recommended [33]. However, RIN values are unfortunately seldom stored in conjunction with the experimental data. The so-called d^k^ degradation index provides a sensitive estimate of RNA-quality that can be computed from raw GeneChip microarray data. It was shown to correlate well with RIN [26]. For fresh tissue, the cutoff of RIN ≥ 7 corresponds to a cut off of the degradation index of d^k^ ≥ 0.45. Note that d^k^ values are not only sensitive, but also specific for RNA quality since only RNA degradation and amplification have such a systematic effect on this value estimated by means of the probe intensity decay with increasing distance between the probe location and the transcription start site [26].

**Figure 1:**
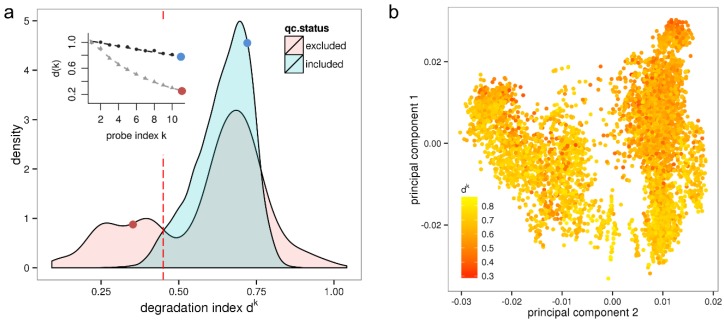
Variation of RNA quality among a large set of microarray samples and its impact on expression results. Panel (**a**) shows the density distribution of d^k^ values measuring the degradation for samples either included or excluded in the HumanExpressionAtlas data set by independent quality control. The red line indicates the low quality threshold corresponding to RIN ≤ 7. The inset shows RNA degradation plots for two selected samples. The corresponding d^k^ values (red and blue dots) are shown in the inset and in the density distribution, respectively; Panel (**b**) shows the first two principal components of the HumanExpressionAtlas expression space where sample points are colored according to their RNA quality measure d^k^.

We have computed the d^k^ values for all 8131 samples of the HumanArraySet which were either included or excluded from the HumanExpressionAtlas data set as described above. Figure 1a shows the resulting density distribution of d^k^ for the qc-included/qc-excluded sample sets. Most samples included after quality control have a degradation index between 0.5 ≤ d^k^ ≤ 0.8 referring to acceptable RNA quality. On the other hand, a large fraction of the qc-excluded samples exhibits values of d^k^ < 0.45 referring to critically low RNA quality. This applies to 25% of the qc-excluded samples and to 10% (868) of all investigated samples. Note that some of these samples might be part of an experiment that is entirely based on low input RNA and multiple rounds of amplification, respectively. Such an experiment can provide valuable information when analyzed individually.

Furthermore, 3% (162) of the qc-included samples are so severely degraded that they should have been excluded by RIN analysis. Expression estimates of these samples are biased, with negative consequences for the reliability of downstream results. That these samples were included in the HumanExpressionAtlas suggests that a more rigorous assessment of RNA-quality should be applied in quality control procedures. Note that our results agrees well with a previous estimation that about 2% of samples of large microarray series have low RNA-quality [34].

Interestingly, only few qc-included samples have d^k^ values larger than 0.8, which obviously represents an upper limit referring to the “weakest possible intensity decay”. This limit can be attributed either to the insufficiency of the clean-up assays to fully stop RNAase activity or, alternatively, to the incomplete amplification of aRNA fragments. On the other hand, a fraction of 8.1% of the qc-excluded samples has values of d^k^ > 0.8 which could be also due to uncertainties in estimating d^k^ at large signal noise levels [26].

We next investigate how the d^k^ parameter changes together with different batches of samples for which we suspect technical variation. To this end, sample groups were defined using a combination of experiment id and experimental date as extracted from the HumanExpressionAtlas. For the experimental date we use the month in which the microarray sample has been hybridized, which can be extracted from Affymetrix raw data files. Supplementary Figure S1 shows the d^k^ parameters for the samples ordered by these batches. Using a simple linear model we obtained a generalized R^2^ statistic with a value R^2^ = 0.67 indicating that a considerable covariation between the batches and the degradation index d^k^.

To test for confounding of the HumanExpressionAtlas with the effect of variable RNA quality we plot its first two principal components of the “expression space” referring to the pre-processed data as available in ArrayExpress in Figure 1b. The same representation was shown in [7] where the data points were however colored according to biological groups. It could be shown that the first principal axis (“hematopoietic axis”) differentiates the biological groups hematopoietic system, solid tissues, connective tissues and incompletely differentiated cell types, and that the second principal axis (“malignancy axis”) differentiates cell lines, neoplasms and normal/non-neoplastic disease tissues. Here, the samples are not colored according to biological groups but instead according to the value of the degradation index d^k^. The resulting plot in Figure 1b shows a clear color gradient along the second principal axis with, in general, lower RNA quality (red spots) at the top and higher RNA quality (yellow spots) at the bottom. The sample groups “normal/non-neoplastic disease tissues” are here associated with better RNA quality and neoplasms with worse RNA quality. Altogether, the correlation coefficient between the degradation index d^k^ and the second principal component is *r* = −0.44. Hence, on average the RNA used for hybridization lost quality with increasing malignancy of the samples.

### 3.2. Assessing RNA Quantity

Before continuing the analysis of potential sources of batch effects, let us first introduce suitable metrics for the assessment of the amounts of RNA available for hybridization in a given sample. To this end, we computed so-called λ and β chip summary measures estimating the relative specific (λ) and non‑specific (β) transcript abundance levels in negative decadic logarithmic scale (see Methods Section) for all samples of the Gene Logic dilution experiment. In this experiment two distinct types of RNA samples, liver tissue and CNS cell line (SNB-19), have been hybridized to Affymetrix Human Genome U95A arrays at varying concentrations [35]. Multiple samples have been prepared from total RNA according to the manufacturers’ protocol and the resulting aRNA has been collected into one master solution for each of the two RNA types. The master solutions, whose RNA concentrations have been determined using an electropherometer (at 260 nm), were then diluted to generate solutions with nominal aRNA masses between 1.25 and 20 µg. Five technical replicates were processed for each concentration, leaving a total of 50 samples.

**Figure 2:**
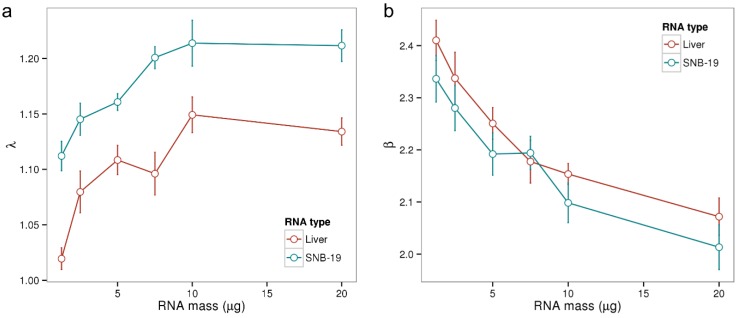
Chip-specific summary parameters λ and β in dependence of the amount of hybridized RNA. We computed both parameters for the samples of Gene Logic’s dilution data set where two types of RNA (liver and SNB-19) have been hybridized at varying concentrations with 5 replicate samples for each concentration. In Panel (**a**) λ increases roughly linear with increasing RNA mass between 1 and 10 µg (Pearson correlations of r > 0.7), but saturates at 20 µg; Panel (**b**) shows how β decreases with increasing RNA mass. An amount of 10 µg aRNA is recommended for the employed HG-U95A platform.

Panel a of Figure 2 displays the obtained λ parameters in dependence of RNA mass for the 50 microarray samples. λ increases with increasing RNA mass between 1 and 10 µg with Pearson correlation coefficients of *r* = 0.71 for liver tissue and *r* = 0.78 for SNB‑19. However, λ does not increase further for a RNA mass of 20 µg which can be explained by the up-down effect: increasing RNA concentrations result in a larger non-specific background accompanied by a smaller effective specific binding constant due to bulk dimerization [21]. The λ summary measure averages the ratio of specifically and non-specifically bound transcripts (see Equation (4)). It is not collinear with RNA mass, as the effect of bulk dimerization is not considered in the hybridization model. In summary, λ describes the amounts of aRNA of a particular microarray hybridization in a non-linear, yet for typical RNA ranges sensitive fashion.

The β parameter from Equation (5) characterizes the dynamic range of the specific hybridization signal (see [5] for a detailed discussion of this chip-specific parameter). As shown in Figure 2b the parameter β decreases with increasing RNA amount. The increasing concentration of RNA in the hybridization solution here results in an increased signal contribution due to non-specific binding and thus in a non-linear, negative effect on the measuring range β.

### 3.3. Amounts of Hybridized RNA

Ideally sufficiently large amounts of aRNA transcripts at constant levels should be used for hybridization to the surface-attached microarray probes to obtain good quality data. In practice this ideal is hard to archive, e.g., due to the considerable variation in the amount of available source RNA. Too high amounts of aRNA can reduce the dynamic range of the fluorescence signals whereas too low amounts increase the signal-to-noise ratio. Consequently, varying RNA amounts can affect gene expression estimates and reduce data quality.

**Figure 3:**
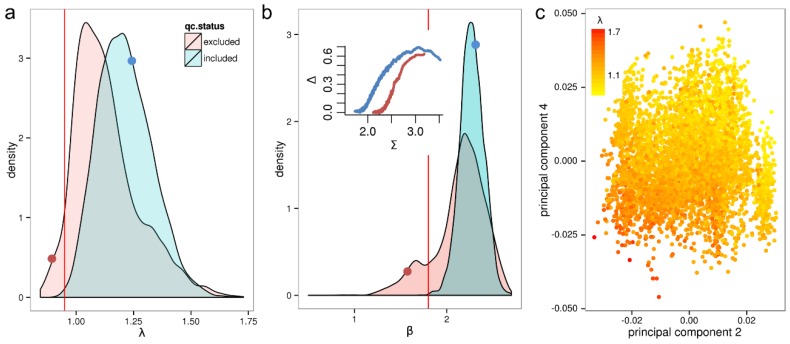
Distribution of the λ and β parameters characterizing the amount of hybridized RNA for qc included/qc-excluded samples (panels (**a**) and (**b**)). The inset shows the hook curves for two selected samples and the corresponding λ and β values (red and blue dots in the same color as the curves). Panel (**c**) shows the principal components two and four of the HumanExpressionAtlas expression space where points are colored according to the value of λ.

The density distribution of the λ parameter is separately shown for the qc-included/qc-excluded sample sets (Figure 3a). For most good quality samples λ ranges between 1.0 and 1.5 with the peak at λ = 1.2. Interestingly, the peak of the distribution is significantly shifted to the left to λ = 1.05 for samples excluded by quality control, showing that low quality samples tend to have decreased relative specific transcript levels (see below).

Virtually none (<0.1%) of the samples that passed stringent quality control exhibit values smaller than λ = 0.95, which we consequently consider a conservative threshold for samples of critically low quality due to decreased specific RNA amounts. We find that 133 (1.6%) of all samples exhibit λ values below this threshold. This equals a fraction of 4.6% from the qc-excluded samples.

The parameter λ describes the average logged specific transcript level in units of the non-specific one. Low expression levels of some genes can have other origins than low RNA amounts, for example local surface deficiencies appearing as weakly shining blurs. Low RNA amounts can also be a result of degradation due to the overlap between the effects of RNA quality and RNA quantity.

The density distribution of the β parameters describing the measurement range of the microarray hybridization is shown in Figure 3b. Low RNA amounts are associated with a larger β values whereas high RNA amounts increase the non-specific background with negative consequences for the measuring range, and thus the signal calibration [5]. For qc-included samples the summary values are distributed closely around the peak at β = 2.25 ± 0.3 whereas for qc-excluded samples the β values spread much broader with a second peak for smaller measuring ranges. Selecting a threshold of β < 1.8, we find that 344 samples (4.2%) have a critically low measurement range.

We also assessed the impact of λ and β by relating them with the first five principal components of the expression space of the HumanExpressionAtlas data set. We obtained a correlation of *r* = −0.61 of λ with the fourth principal component (see Figure 3c). Furthermore, a correlation of *r* = −0.33 with the second principal component, which we previously showed to relate with RNA quality, was found. The other three components show only low correlations of −0.16 < *r* < 0.21. With coefficients of −0.04 < *r* < 0.01, the β parameter exhibits no correlation with the first five principal components. In summary, the relative specific signal shows significant systematic effects on the expression measures whereas the amount of non-specific hybridization does essentially not.

### 3.4. Sequence Effect Size

Nucleic acid folding and formation of DNA/DNA or DNA/RNA duplexes on surfaces are fundamental reactions for any microarray assay and largely depend on the hybridization conditions. For example, the temperature and ion composition of the hybridization reactions can affect nucleic acid binding [36]. Different hybridization conditions can lead to sequence-dependent variations in the probe intensity signals, which can further propagate to the gene expression estimates and therefore constitute potential artifacts.

We assess the sequence effect using the parameter log(K_diff_) referring to the maximum pairwise difference between all positional-dependent NN-sensitivity profiles (see Methods Section 2.3). For example, a value of log(K_diff_) = 5 for a particular hybridization means that, on the average, two hypothetical probes (most likely with the sequences AAA…A and CCC…C) would differ in their intensity values by 5 orders of magnitude. log(K_diff_)can thus be thought of as the maximum strength, or impact, of the sequence effect.

**Figure 4:**
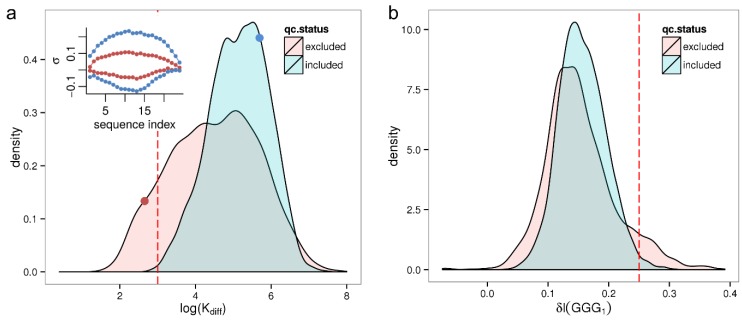
Distribution of summary parameters related to sequence effects for qc-included/qc-excluded samples of the HumanExpressionAtlas. Panel (**a**) shows the parameter log(K_diff_) as a measure of the total strength of the sequence effect. The inset shows the corresponding sequence profiles for two selected samples (red and blue dots, in the same color as the profiles); Panel (**b**) shows the intensity increase due to the (GGG)_1_ motif, δI(GGG)_1_.

As previously we computed log(K_diff_) for all 8131 samples of the HumanArraySet plotting the respective density distribution in Figure 4a. By trend qc-excluded samples show a lower maximum sensitivity amplitude, rendering it a potential marker for low quality samples. Based on the observation that barely any good quality samples (<0.1%) exhibit a smaller maximum sensitivity amplitude, log(K_diff_) = 3 is chosen as conservative threshold selecting samples with critically low sequence effect size. This applies to a fraction of 4.1% of all samples.

The largest correlation of the log(K_diff_) parameter with the first five principal components of the HumanExpressionAtlas (in absolute scales) is *r* = −0.17 with the third principal component. Correlations for the remaining principal components are smaller than |r| = 0.11. In conclusion, the sequence effect size is not a technical variable with a large impact on the most common patterns in the expression space.

### 3.5. Guanine Effects

It was previously found that runs of guanines within the probe sequence, particularly runs of guanines as long or longer than three, significantly affect the obtained signal intensities [34]. We showed that this (GGG)_1_ effect propagates through the various preprocessing methods of microarray data analysis and can lead to biased expression estimates [25]. The origin of the (GGG)_1_ effect lies in the formation G-quadruplex structures. The formation of duplexes between negatively charged nucleic acids in general, and between G-quadruplexes in particular, depends on the ionic strength and thus on the employed solution buffer [37]. This dependency on the ionic strength also applies to hybridization reactions on solid surfaces [38].

We here define the strength of the guanine effect in terms of the *intensity increase due to the (GGG)_1_ motif* as follows

(6) $$\delta I({\text{GGG}}_{1})={\langle {\text{logI}}_{p}\rangle }_{\xi_{p}^{1,3}=(\text{GGG})}-{\langle {\text{logI}}_{p}\rangle }_{{(\text{TTT})\in \xi_{p}}^{\cdot }}$$

A value of δI(GGG)_1_ = 0.3 thus reflects an on the average 10^0.3^ ≈ 2 times as large intensity of probes containing the (GGG)_1_ motif compared to probes containing (TTT) anywhere in their sequence. The average intensity of (TTT) containing probes here serves as appropriate baseline.

Figure 4b shows the density distribution of the δI(GGG)_1_ parameter which varies between 0 < δI(GGG)_1_ < 0.3 for qc-included samples. We consider samples with a threshold of δI(GGG)_1_ > 0.25 to be significantly affected by GGG_1_ effect—this reflects an intensity increase of +75% and would lead to a significant bias in the expression estimates of transcripts being targeted by the respective probes. Accordingly, 254 (3.1%) of the samples have a GGG_1_ related intensity bias. Many of them are removed by strict quality control; only 63 (1.1%) of qc-included samples are above the threshold.

Assessing the correlation of δI(GGG)_1_ with the first five principal components of the HumanExpressionAtlas expression data we observe correlation coefficients between 0.14 < |r| < 0.19. Consequently, guanine effects have only a minor impact on the common patterns in the expression space. Note that the RMA method was used for the preprocessing of the HumanExpressionAtlas calibration, and we showed previously that its expression estimates are in general susceptible to GGG_1_ effects [25]. 

### 3.6. Discussion

We have studied the general prevalence and impact of a RNA quality, RNA quantity and sequence effects using a large and representative set of microarray samples from the Affymetrix HG-U133a platform. We defined parameters, or used previously defined ones, that quantify each technical artifact based on systematic changes in the microarray intensity signals. We determined appropriate thresholds indicating samples of questionable quality, which associate with or even potentially cause biased expression estimates due to the respective factors. The impact of the technical variables on the expression estimates was analyzed by computing correlations with the first five principal components of the expression space of the HumanExpressionAtlas. The results are summarized in Table 1.

**Table 1:** Prevalence and impact of technical factors that constitute potential sources of batch effects in gene expression experiments. The second column shows the percentage of samples that are critically affected by the respective technical artifact. The selection of appropriate thresholds is reasoned in the respective subsections. Column two denotes the prevalence among all samples in the HumanArraySet, in column three among the subset of samples that have been selected after quality control independently performed by [7]. Column four shows the correlation of the technical variable with the principal components of the expression space, and last column how it changes along with known batches.

Parameter	Prevalence (all)	Prevalence (qc Included)	Correlation with Principal Component (pc)	Adjusted R2 with Batch (Lm)
Degradation index d^k^	10.6%	3.0%	−0.44 (2)	0.67
Specific transcript level λ	1.6%	(0.1%)	−0.61 (4)	0.62
Measuring range β	4.2%	(0.1%)	0.04 (1)	0.24
Sequence effect size	4.1%	(0.1%)	−0.17 (3)	0.76
Guanine effects	3.1%	1.1%	0.19 (5)	0.76

We found that a large fraction of 10% of the 8131 samples are so severely degraded that they should be excluded from further analysis. While most of these samples where indeed excluded from the HumanExpressionAtlas, further 3% of the low-quality RNA samples passed quality control, highlighting the need for a more rigorous assessment of RNA quality in microarray data analysis.

A fraction of 1.6% of the samples has decreased specific transcript levels λ, relating to low amounts of hybridized RNA. Samples excluded by quality control in general have smaller λ values. Analysis of the β parameter shows that about 4% of the samples have low measurement ranges and that β has almost no impact on the predominant variation patterns in the expression space.

Importantly, RNA quality and RNA abundance variation were found to have potentially an unexpectedly high impact on the gene expression results. Both technical variables strongly correlate with the most common patterns of expression variation in the HumanExpressionAtlas. The RNA quality measure d^k^ correlates with the second principal component for which previously a biological interpretation was found. The relative specific transcript level parameter λ is highly correlated with another principal component. Together with the observed high prevalence of these artifacts, these technical factors thus constitute major sources of batch effects.

We found that sequence effects are highly variable in the investigated Affymetrix HG-U133a platform. The sequence effect size is particularly low among low-quality samples where 4% of the samples are affected. 3% of the samples have a strong guanine-run (GGG_1_) effect with critical impact on some transcripts [25]. They constitute an important technical artifact, which, however, only affects the expression estimates of some genes in some of microarray samples. It obviously does not affect the majority of features and is consequently not a major determinant of systematic variation in the expression space. The overall impact of the studied sequence effects on the expression space is relatively low.

The term “technical artifact” used in this publication refers to all factors which cause improperly calibrated expression measures which then do not properly reflect the amount of mRNA in the object of study [39]. Recall that expression microarrays were designed to measure the relative abundance of mRNA in the samples of interest in a gene- (or exon-) specific fashion. Consequently, correct expression measures by definition should be independent of characteristics such as RNA quality or RNA quantity used for hybridization and also of probe sequences interrogating the respective gene. On the one hand, technical artifacts can be caused by improper sample handling (e.g., by using degraded mRNA or an unsuitable amount of mRNA) or improper instrument settings (e.g., of the scanner or fluidic station). On the other hand, technical artifacts can be inherent in the measuring principle of the microarray technology due to surface hybridization effects not related to transcript abundance, even if samples and instruments were handled ideally. That means that also “biological” effects such as the massive changes of transcript composition in the cell and/or of their total abundance level e.g., with changing malignancy can cause systematic variations of quality measures. Although of biological origin we also assign such variations as “technical’ effects because they are related to unwanted side effects of the microarray technology. We have shown in our previous work (see Methods Section) that these side effects can cause systematic errors of the gene-related expression values with potential impact for the interpretation of the studies in terms of biological function. Hence, the identification of batches of samples differing in their technical characteristics potentially reflects improper calibration and in final consequence biased expression measures.

In this publication we used the correlation of selected quality measures with the principal components of the expression data as a surrogate measure of the resulting bias, which reflects associations between the expression measures and the respective technical factor. In worst case, this observation indicates that the change of expression values described by the principal component(s) is affected or even determined by systematic errors caused by the respective technical factors. In best case, the gradient of expression values is “correct” in the sense that it is not affected by the technical factors. In this case the associated gradient of quality measures can reflect an independent variation of the respective technical parameters, which can have their origin also in biological factors such as the malignancy of the tumor samples. In the former situation the expression values and their interpretation is questionable whereas the latter situation potentially indicates unexpected and possibly interesting systematic changes of biological factors such as global changes of total expression level in the cells which, for example, can shift the relation between absent and present genes. Unfortunately the “worst” case is much more common according to our experience and it is hard to explain what, e.g., the Guanine-effect or truncated transcripts have to do with changes of transcriptional regulation. On the other hand, the second situation cannot be excluded owing to interferences between the different quality measures and/or with other, still unknown biological factors.

A more detailed analysis should extract consequences on gene level to identify the particular causes of the observed biases of gene expression and of the quality measures in the samples studied. Such forthcoming studies are of high interest because they not only can help to improve the calibration of microarrays. They will also enable new insights into biological mechanisms underlying the observed biases such as the systematic variation of the total expression level as a function of biological factors.

Methods to gradually correct these biases are required at the level of data analysis. A natural first choice are the existing batch effect removal methods (see [4] for a review) which can help significantly reducing technical variation. These methods rely on batch information and thus cannot cope with covariation patterns more complex than available surrogates/sample groupings. For some covariates, additional parameters can be estimated using physico-chemical measures of the microarray hybridization. This can also be used to correct specific technical effects as we have shown previously for RNA degradation and sequence effects (see [5,25,40] for discussion and software tools). However, such methods are currently only available for a limited set of expression quantification technologies such as the GeneChip expression arrays.

It is therefore of great importance to consider the downstream effects of the unavoidable technical variation already in the lab. Recording and storing independent measures for technical parameters such as RNA quality and quantity should be mandatory for all processed samples. Only with the help of this meta-data known technical variation can be reliably separated from biological variation in large-scale expression studies and integrative analyses. It is highly probable that these factors affect not only the GeneChip microarrays investigated here but also other gene expression technologies such as RNA-seq for which only recently quality control measures have been established [41].

## 4. Conclusions

In prospect of the presented results it becomes clear that biological interpretations based on expression changes in large data sets might be misleading because they potentially interfere with technical effects. In particular variation of RNA quality and RNA quantity constitute major sources of technical bias and should be considered in every large-scale gene expression study as well as in integrative analyses of combined data sets.

What can be done to alleviate unwanted technical variation and its effects? The importance of quality control for microarray gene expression experiments is already well accepted—partly thanks to initiatives such as the MAQC. Independent measurements, for example of RNA quality, are de-facto standard protocol. These measures ensure that all samples fulfill at least a minimum quality criterion prior to microarray hybridization. We here however showed how several technical factors affect expression results to a degree that goes beyond simple good-quality *vs*. bad-quality decisions. We therefore encourage that technical variation be explicitly considered at the various steps of a microarray experiment (during sample preparation and microarray hybridization in the lab as well as during data analysis procedures) as part of a comprehensive quality control.

## Supplementary Files

Supplementary File 1
