# Supplementary material for: Variation of RNA Quality and Quantity Are Major Sources of Batch Effects in Microarray Expression Data
Source: Microarrays (Basel). 2014 Dec 16;3(4):322–39. doi: 10.3390/microarrays3040322 (PMC4979052; doi:10.3390/microarrays3040322)

Supplementary Materials

Variation of RNA Quality and Quantity Are Major Sources of Batch Effects in Microarray Expression Data

Mario Fasold and Hans Binder

1. Covariation of Technical Variables and Batches

Figure S1. Chip summary parameters computed for samples of the HumanExpressionSet. Samples have been assigned, and ordered by, batches defined as experimental name and month of hybridization. Black lines denote borders between the sample groups. For convenience, only groups with at least 20 samples per group are shown here.

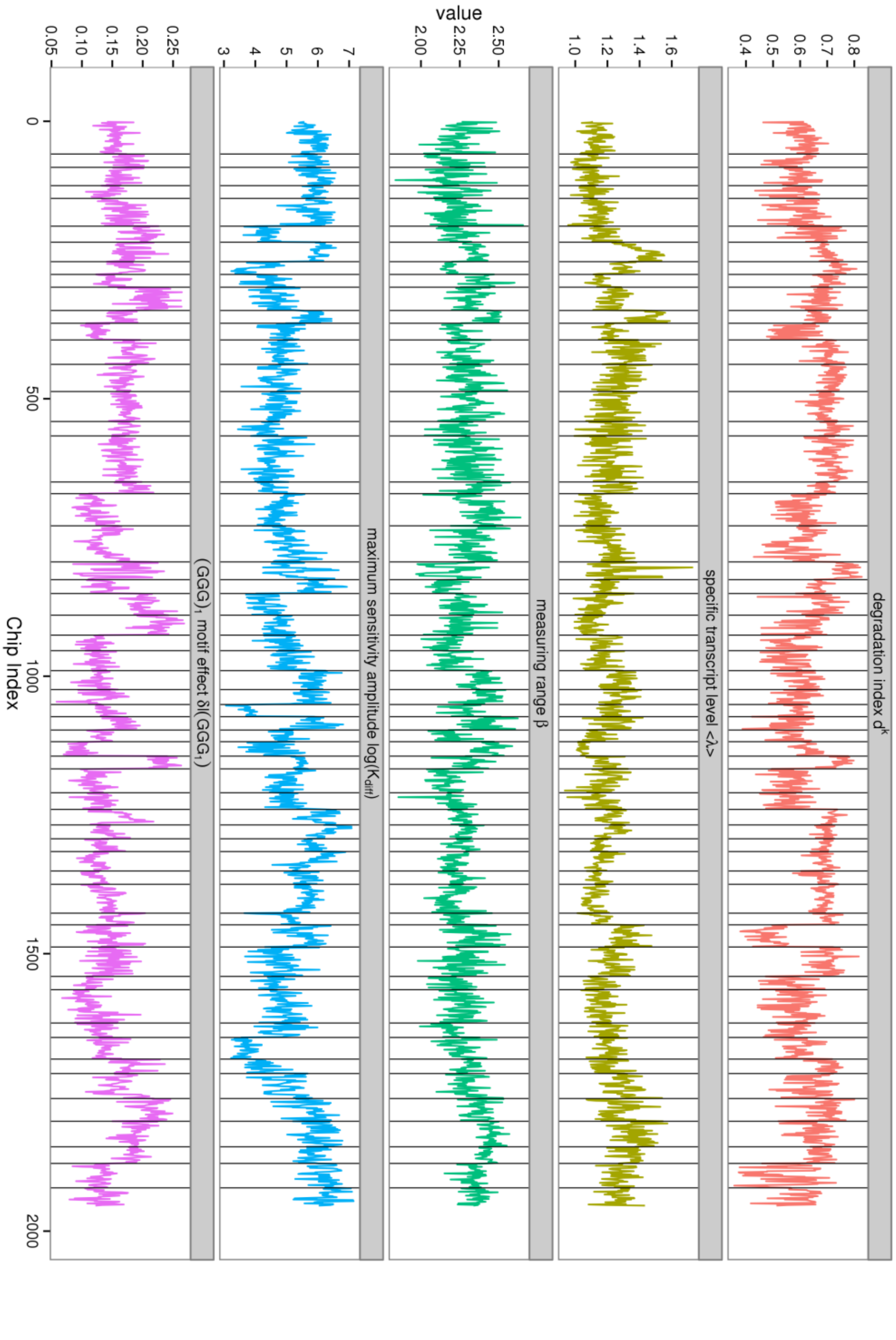

2. Sensitivity Profiles

Figure S2. Typical sensitivity profiles (r = 2, positional-dependent nearest neighbor model) of an Affymetrix HG-U133a microarray. Points indicating positional-dependent sensitivity terms for four selected base tuples (AA,CC, GC, GG) are shown in black, whereas the points and connecting lines of the remaining 12 base tuples are shown in gray.

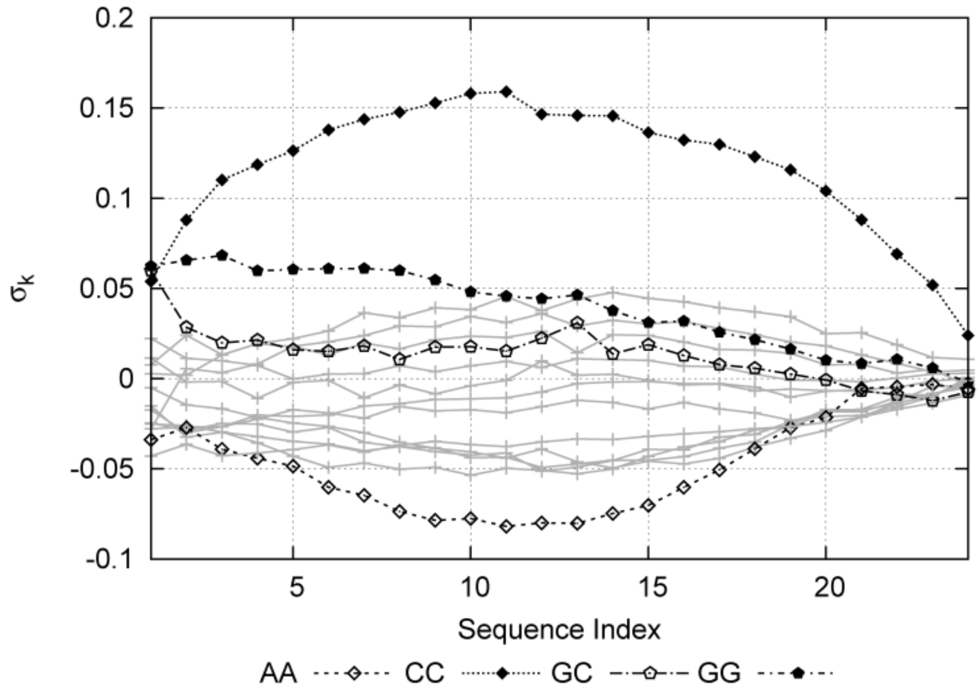

Supplement: Supplementary File 1 [file microarrays-03-00322-s001.pdf]
